# Functional capillary impairment in patients with ventricular assist devices

**DOI:** 10.1038/s41598-019-42334-3

**Published:** 2019-04-11

**Authors:** Patricia P. Wadowski, Barbara Steinlechner, Daniel Zimpfer, Thomas Schlöglhofer, Heinrich Schima, Martin Hülsmann, Irene M. Lang, Thomas Gremmel, Renate Koppensteiner, Sonja Zehetmayer, Constantin Weikert, Joseph Pultar, Bernd Jilma

**Affiliations:** 1 Department of Internal Medicine II, Division of Angiology, Medical University of Vienna, Vienna, Austria; 2 Division of Cardiothoracic and Vascular Anesthesia, Medical University of Vienna, Vienna, Austria; 3 Department of Surgery, Division of Cardiac Surgery, Medical University of Vienna, Vienna, Austria; 4 Department of Medical Physics and Biomedical Engineering, Medical University of Vienna, Vienna, Austria; 5 Ludwig-Boltzmann-Cluster for Cardiovascular Research, Vienna, Austria; 6 Department of Internal Medicine II, Division of Cardiology, Medical University of Vienna, Vienna, Austria; 7 Intitute of Medical Statistics, Medical University of Vienna, Vienna, Austria; 8 Department of Clinical Pharmacology, Medical University of Vienna, Vienna, Austria

## Abstract

The implantation of continuous – flow ventricular assist devices (VAD) is suggested to evoke angiodysplasia contributing to adverse events such as gastrointestinal bleeding. We evaluated *in vivo* capillary density and glycocalyx dimensions to investigate possible systemic microvascular changes in patients with chronic heart failure and VAD support vs. standard medical treatment. Forty-two patients with VAD support were compared to forty-one patients with ischemic and non-ischemic chronic heart failure (CHF) on standard pharmacotherapy and to a group of forty-two healthy subjects in a prospective cross-sectional study. Sublingual microcirculation was visualized using Sidestream Darkfield videomicroscopy and functional and perfused total capillary densities were quantified. Patients with VAD implantation were followed for one year and bleeding events were recorded. Median time after VAD implantation was 18 months. Patients were treated with centrifugal-flow devices (n = 31) or axial-flow devices (n = 11). Median functional capillary density was significantly lower in patients with VAD therapy as compared to CHF patients (196 vs. 255/mm^2^, p = 0.042, adjusted p-value). Functional and total capillary densities were 44% and 53% lower (both p < 0.001) in patients with VAD therapy when compared to healthy subjects. Cox regression analysis revealed loss of capillary density as a significant predictor of bleeding events during one -year follow-up of VAD patients (HR: 0.987, CI (95%): 0.977–0.998, p = 0.021 for functional and 0.992, CI (95%): 0.985–0.999, p = 0.03 for total capillary density). In conclusion, patients with VAD support exhibit capillary density rarefaction, which was associated with bleeding events. If confirmed independently, capillary impairment may be evaluated as novel marker of bleeding risk.

## Introduction

Continuous-flow ventricular assist devices (VADs) are increasingly implanted for long-term therapy in patients with advanced heart failure^1^. Though the hemodynamics of the large vessels are greatly improved by VAD in heart failure patients as a result of the mechanical circulatory support, flow pattern changes potentially evoke stasis and the risk of pump thrombosis and stroke^2,3^. In addition, acquired von Willebrand syndrome (AVWS) develops due to altered hemodynamic conditions^4,5^. It results from the loss of high molecular weight multimers of von Willebrand factor (vWF) caused by enhanced shear forces and is dependent on platelets and ADAMTS - 13^6,7^. In patients with VAD treatment, adverse events such as bleeding, pump thrombosis and stroke are associated with altered hemodynamic conditions and biocompatibility^8–10^. In particular, the development of an AVWS in VAD patients may be responsible for angiodysplasia^11^ and promote non-surgical bleeding^12,13^.

Therefore, we hypothesized that rheologic alterations following the implantation of continuous-flow VAD - despite macrovascular hemodynamic improvement - might be associated with systemic changes in microvascular hemodynamics, which could be visualized by *in vivo* sublingual videomicroscopy.

## Methods

### Ethical approval

The study was performed in accordance with the Declaration of Helsinki and approved by the local Ethics Committee of the Medical University of Vienna (EC- Nr: 1734/2013). All participants provided written informed consent.

### Study population

This single-center prospective cross-sectional study was performed in forty-two patients with end stage heart failure and VAD implantation (VAD group). As control groups, we enrolled forty- one stable outpatients with a history of severe systolic chronic heart failure (CHF group) under guideline- directed medication^14^ and forty-two healthy subjects (healthy controls group). Patients with VAD support were included consecutively during their routine outpatient visits.

The CHF group was defined as follows: confirmed heart failure with reduced ejection fraction (below 40%)^14^ and at least one finding of NT-proBNP level above 2000 pg/ml in one of the preceding clinical visits.

Prescribed medication was continued and patients were measured after fasting overnight. Outpatient visits of patients with VAD treatment were scheduled every three months for one year. We recorded current medication, age and weight/height and obtained routine laboratory parameters on the same day as the videomicroscopy in all study participants.

### Microscope imaging

*In vivo* measurements of the sublingual vasculature were performed as published previously^15^ using a sidestream darkfield videomicroscope (CapiScope HVCS Handheld Video Capillary Microscope, KK Technology, England) by one person to avoid inter-observer variability. The camera is equipped with light emitting diodes that use a wavelength of 525 nm to detect the hemoglobin of circulating red blood cells. The standard lens of the microscope enables a 0.92 µm/pixel magnification in 752 × 480 pixels (field of view: 692 × 442). The software for acquisition and calculation of the perfused boundary region (PBR) was supplied by GlycoCheck BV (Maastricht, The Netherlands) and detailed methodology was described by Lee *et al*.^16^. In short, the camera was placed under the tongue near the frenulum and the software identified micro-vessels below 30 µm of thickness due to contrast of red blood cells (RBC). RBC column widths were measured in at least 3000 vessel segments. The PBR is the most luminal part of the glycocalyx, which allows for limited penetration of the RBCs^17^. It is located at both sides of the RBC column; to determine its properties, the distance between the median RBC column width (P50) and the outer edge of the RBC- perfused luminal part of the glycocalyx (=perfused diameter) was calculated using the following equation: (perfused diameter-median RBC column width)/2. The increase in PBR reflects glycocalyx destruction^15–18^. The average PBR of microvessels between 5–25 µm diameter was used for statistical analyses. Method validation of PBR measurements was described by Dane *et al*.^18^: it was shown that changes in PBR dimensions are reflected by RBC column size and independent of vascular diameter; PBR is inversely proportional to the glycocalyx^18^.

To assess capillary density, the software recognized all micro-vessels below 30 µm of thickness by determination of the red blood cells against the background. Vascular segments (line markers) were placed every 10 µm of the vessel length. The recording process continues until a minimum of 3000 vascular segments have been measured. After acquisition, a total of 21 line markers were placed in intervals of 0.5 µm of the vascular segments on the first frame of each recording session. Only those vessels with an appropriate contrast of more than 60% of all 21 line markers were considered as functional (=valid perfused) vessels. All perfused vessels are referred to as total capillary density. RBC filling percentage is calculated by determining the percentage of vessels with RBCs present during the recording session (corresponding to 40 frames per session)^16^. RBC filling percentage and perfused capillary density are regarded as estimates of microcirculatory perfusion^15,16^.

### Sample size calculation and Statistical analysis

We hypothesized a minimum 18% difference in capillary density between CHF patients and controls referring to a previous publication^19^. Based on our preliminary assessment of the variability of capillary density in normal controls (CV = 24%), we estimated that we would need to include in total 87 participants to account for comparisons between multiple groups (n = 3). We decided to recruit relatively more patients to compensate for potential technical problems and because we anticipated a larger variability in the CHF patients compared to controls^19^.

We decided to exclusively perform non-parametric testing due to outliers in the microvascular data and skewed distributions. Groups were compared with the nonparametric Mann- Whitney- U- Tests, or χ^2^ analyses, as appropriate. The primary research question was the comparison between the VAD and CHF group as well as between the VAD group and healthy volunteers, as differences between CHF patients and healthy volunteers were already described^15^. P-values were therefore adjusted by the Bonferroni correction for two comparisons.

Vascular parameters (PBR, RBC filling percentage, perfused and total capillary density) and all continuous variables are presented as median and interquartile range. Correlations between microcirculatory data and VAD parameters were calculated using Spearman’s rank correlation. Two-sided p-values < 0.05 were considered statistically significant. A logistic regression analysis was performed to describe the relationship between functional or total perfused capillary density and bleeding events and the odds ratio as well as the 95% confidence intervals (CI) were calculated. Cox regression analyses were performed to assess the hazard ratio to develop bleeding events during one-year follow-up in VAD patients.

In addition, receiver-operating characteristic (ROC) curve analyses were performed including standard error (SE) and 95% confidence intervals (CI) and used to graphically depict the relation between bleeding events and perfused capillary density as well as for calculation of predictive thresholds for capillary density with respect to bleeding events.

Statistical analyses were performed using IBM SPSS Statistics for Macintosh, Version 21.0. (IBM Corp. Armonk, NY, Released 2012).

## Results

### Clinical characteristics

Detailed characteristics of all study participants are depicted in Table 1. Twenty-six patients with non-ischemic and 16 patients with ischemic cardiomyopathy were recruited into the VAD group. Median time after VAD implantation was 18 months (6–29 months).

**Table 1:** Clinical characteristics of study participants.

	VAD patients (n = 42)	CHF patients (n = 41)	Healthy controls (HC) (n = 42)	P – value between	
Age, years	61 (53–69)	66 (58–72)	65 (57–73)	p = 0.128*	VAD/CHF
				p = 0.270*	VAD/HC
Male Sex, n	33 (79%)	37 (90%)	28 (67%)	p = 0.288*	VAD/CHF
				p = 0.442*	VAD/HC
Body mass index	27 (24–31)	29 (25–33)	25 (24–28)	p = 0.372*	VAD/CHF
				p = 0.098*	VAD/HC
Serum creatinine (µmol/L)	99.9 (80–133.5)	114.5 (95.3–187.2)	80 (70.9–89.3)	p = 0.046*	VAD/CHF
				p < 0.001*	VAD/HC
Estimated glomerular filtration rate (ml/min)	64.3 (48.5–88)	55.5 (29.8–73.4)	79.5 (69.1–94.7)	p = 0.094*	VAD/CHF
				p = 0.006*	VAD/HC
C-reactive protein (mg/L)	6.3 (2.5–10.8)	4.2 (1.6–8.2)	1.5 (0.9–2.5)	p = 0.346*	VAD/CHF
				p < 0.001*	VAD/HC
Fibrinogen (g/L)	4.09 (3.64–4.62)	4.15 (3.59–4.55)	3.33 (2.90–3.89)	p = 1*	VAD/CHF
				p < 0.001*	VAD/HC
Leukocytes (*10^9^/L)	8.1 (6.7–10.1)	7.8 (6.1–9.2)	6.2 (5.6–7.8)	p = 0.592*	VAD/CHF
				p < 0.001*	VAD/HC
Platelets (*10^9^/L)	233 (197–271)	205 (165–241)	235 (209–252)	p = 0.054*	VAD/CHF
				p = 1*	VAD/HC
Total bilirubin (µmol/L)	11 (6.9–16.2)	10.1 (6.7–20.7)	7.2 (5.3–9.8)	p = 1*	VAD/CHF
				p = 0.014*	VAD/HC
International Normalized Ratio	2.7 (2.3–3)	1.3 (1–2.7)	1.00 (0.9–1.00)	p = 0.002*	VAD/CHF
				p < 0.001*	VAD/HC
NT-proBNP (ng/L)	1625 (819–3177)	2522 (1483–4298)		p = 0.053	VAD/CHF

Data are presented as median and IQR. *Adjusted p -values.

Medication of the VAD and CHF group is described in the supplement, Table 1. Patients with VAD treatment were aged between 35 and 76 years and CHF patients between 25 and 77 years. Overall, 31 patients were treated with centrifugal-flow devices (HeartWare® Ventricular Assist System (HVAD®, Medtronic Inc., USA)) and 11 patients with axial-flow devices (Heartmate II, Abbott, North Chicago, USA). VAD patients had a pump flow of 5.2 L/min (4.5–5.9 L/min), which corresponds to almost normalized levels of cardiac output.

Seventeen patients with non-ischemic and 24 with ischemic heart failure were included into the CHF group. Clinically, 5% of the VAD and 18% of the CHF patients were NYHA stage I, 48% of the VAD and 36% of the CHF patients NYHA stage II, 45% of the VAD and 46% of the CHF patients NYHA stage III, and 2% of the VAD and none of the CHF patients NYHA stage IV (p = 0.19). There was no difference in age between VAD and CHF patients or healthy controls, Table 1. VAD patients had significantly lower creatinine levels compared to the CHF group. Due to mandatory oral anticoagulation the INR was higher in patients on VAD therapy compared to the CHF group and healthy controls. Several disease-related variables were different between VAD patients and healthy volunteers: serum creatinine and estimated glomerular filtration rate, CRP, fibrinogen, leukocytes, total bilirubin and INR.

### Decreased capillary density in patients with VAD therapy

**Figure 1:**
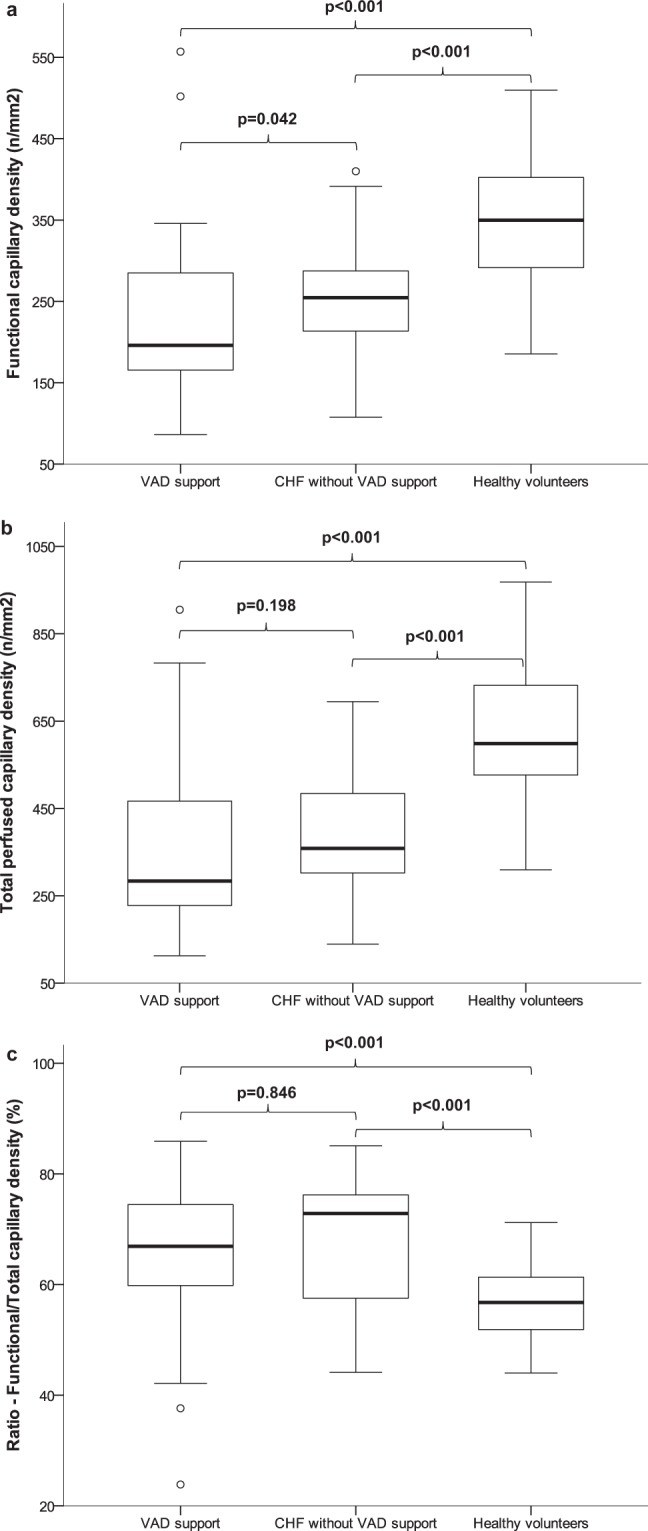
(**a**) Functional capillary density in chronic heart failure patients with and without VAD support. (**b**) Total perfused capillary density in chronic heart failure patients with and without VAD support. (**c**) Ratio of functional capillary density/total perfused capillary density in chronic heart failure patients with and without VAD support. The boundaries of the box show the lower and upper quartiles of data, the line inside the box represents the median. Whiskers are drawn from the edge of the box to the highest and lowest values that are outside the box but within 1.5 times the box length. The outliers are presented as open circles. CHF, chronic heart failure; VAD, ventricular assist device.

Patients on VAD therapy had significantly lower functional (196/mm^2^ (165–286/mm^2^) vs. 350/mm^2^ (292–403/mm^2^), p < 0.001, adjusted p-value) and total perfused capillary density (284/mm^2^ (228–473/mm^2^) vs. 599/mm^2^ (525–740/mm^2^), p < 0.001, adjusted p-value) in comparison to healthy subjects, Fig. 1a,b. Despite beneficial effects on macrovascular hemodynamics as monitored by VAD parameters, there was a further significant decrease in functional capillary density in comparison to the CHF group (196/mm^2^ (165–286/mm^2^) vs. 255/mm^2^ (212–288/mm^2^), p = 0.042, adjusted p-value), Fig. 1a. Total perfused capillary density did not differ between the VAD and CHF group (284/mm^2^ (228–473/mm^2^) vs. 359/mm^2^ (302–490/mm^2^), p = 0.198, adjusted p-value), Fig. 1b. As described previously^15^, there was also in this analysis a significant difference in functional and total capillary density between CHF patients and healthy controls (p < 0.001, respectively, unadjusted), Fig. 1a,b.

The ratio of functional capillary density/total perfused capillary density was higher in VAD and CHF patient groups than in healthy subjects (patients with VAD treatment: 67% (60–75%), CHF patients: 73% (57–77%) vs. healthy controls: 57% (51–61%), p < 0.001, adjusted p-value, respectively). However, it was similar between CHF patients with and without mechanical circulatory support, Fig. 1c.

There were no differences in capillary density between ischemic or non-ischemic cardiomyopathy in VAD patients (Table 2) and in CHF patients (data not shown).

**Table 2:** Microcirculatory parameters in VAD- treated patients by disease etiology.

	Non-ischemic CMP (n = 26)	Ischemic CMP (n = 16)	p-value
Functional capillary density (n/mm^2^)	182 (145–271)	202 (184–296)	0.271
Total capillary density (n/mm^2^)	274 (217–477)	317 (247–483)	0.453
Ratio (%) (Functional/Total capillary density)	70 (60–75)	66 (60–70)	0.312
Perfused boundary region (µm)	1.89 (1.75–2)	1.99 (1.87–2.2)	0.06

Data are presented as median and IQR.

### Glycocalyx thickness in patients and healthy volunteers

The perfused boundary region (PBR) was similar between both patient groups and healthy volunteers: 1.92 µm (1.76–2.06 µm) in VAD patients vs. 1.97 µm (1.70–2.07 µm) in CHF patients and 1.85 µm (1.76–2.01 µm) in healthy controls (all p > 0.05, respectively). Glycocalyx dimensions were 5% lower in VAD- treated patients with ischemic cardiomyopathy compared to non-ischemic CMP. However, this was not statistically significant, Table 2.

In line with previous studies, there was a high negative association between PBR and RBC filling in all groups (patients with VAD support: r = −0.67; CHF without VAD therapy: r = −0.91, healthy subjects: r = −0.77, all p < 0.001) indicating a link between PBR and microcirculatory perfusion^15,16^.

### Relationship between functional capillary density and events in patients on VAD therapy

During the one-year follow up, eleven VAD patients experienced bleeding events (gastrointestinal bleeding: n = 7, gastrointestinal and cerebral bleeding: n = 1, cerebral bleeding: n = 1, epistaxis = 2). All except one bleeding event required blood transfusion or were considered life-threatening. At baseline, patients with bleeding events showed lower INR (2.2 [1.5–2.6] vs. 2.8 [2.5–3], p = 0.018). At the time of the bleeding events, these patients were on phenprocoumon and only 7 patients were on aspirin (median 100 mg/day, IQR: 0–100 mg/day). One of the patients was over-anticoagulated during septic state (INR above 7.5), whereas the other patients were in the range of advised anticoagulation (INR 2.1 [1.9–2.9]). According to the ISHLT (The International Society for Heart and Lung Transplantation) Guidelines, an INR between 2.0–3.0 has to be achieved, as recommended by device manufacturers^20^.

Patients with bleeding events at follow- up had significantly lower functional (146/mm^2^ (89–200/mm^2^) vs 216/mm^2^ (175–300/mm^2^), p = 0.006) and total perfused capillary density (228/mm^2^ (147–272/mm^2^) vs 346/mm^2^ (248–502/mm^2^), p = 0.005) compared to non-bleeding VAD patients, Table 3. The odds ratio was 0.983, CI (95%): 0.97–0.997), p = 0.019, for functional capillary density and 0.992, CI (95%): 0.985–0.999, p = 0.035 for total perfused capillary density; the results are in accordance with Table 3. The hazard ratio to develop bleeding events during the one- year follow up was 0.987, CI (95%): 0.977–0.998, p = 0.021 for functional capillary density and 0.992, CI (95%): 0.985–0.999, p = 0.03 for total perfused capillary density as calculated by the cox regression analysis.

**Table 3:** Microcirculatory parameters in patients with bleeding events during the one-year follow up.

	Bleeding events (n = 11)	No bleeding events (n = 31)	p-value
Functional capillary density (n/mm^2^)	146 (89–200)	216 (175–300)	0.006
Total capillary density (n/mm^2^)	228 (147–272)	346 (248–502)	0.005
Ratio (%) (Functional/Total capillary density)	74 (59–77)	67 (60–73)	0.498
Perfused boundary region (µm)	1.92 (1.70–2.21)	1.91 (1.77–2.05)	0.888

Data are presented as median and IQR.

For every increase in capillary density per quartile, there is a relative bleeding risk reduction of more than 50%: HR = 0.483, CI (95%) = 0.254–0.921, p = 0.027 for functional capillary density and HR = 0.489, CI (95%) = 0.257–0.929, p = 0.029 for total capillary density. The quartiles are given in the supplement.

**Figure 2:**
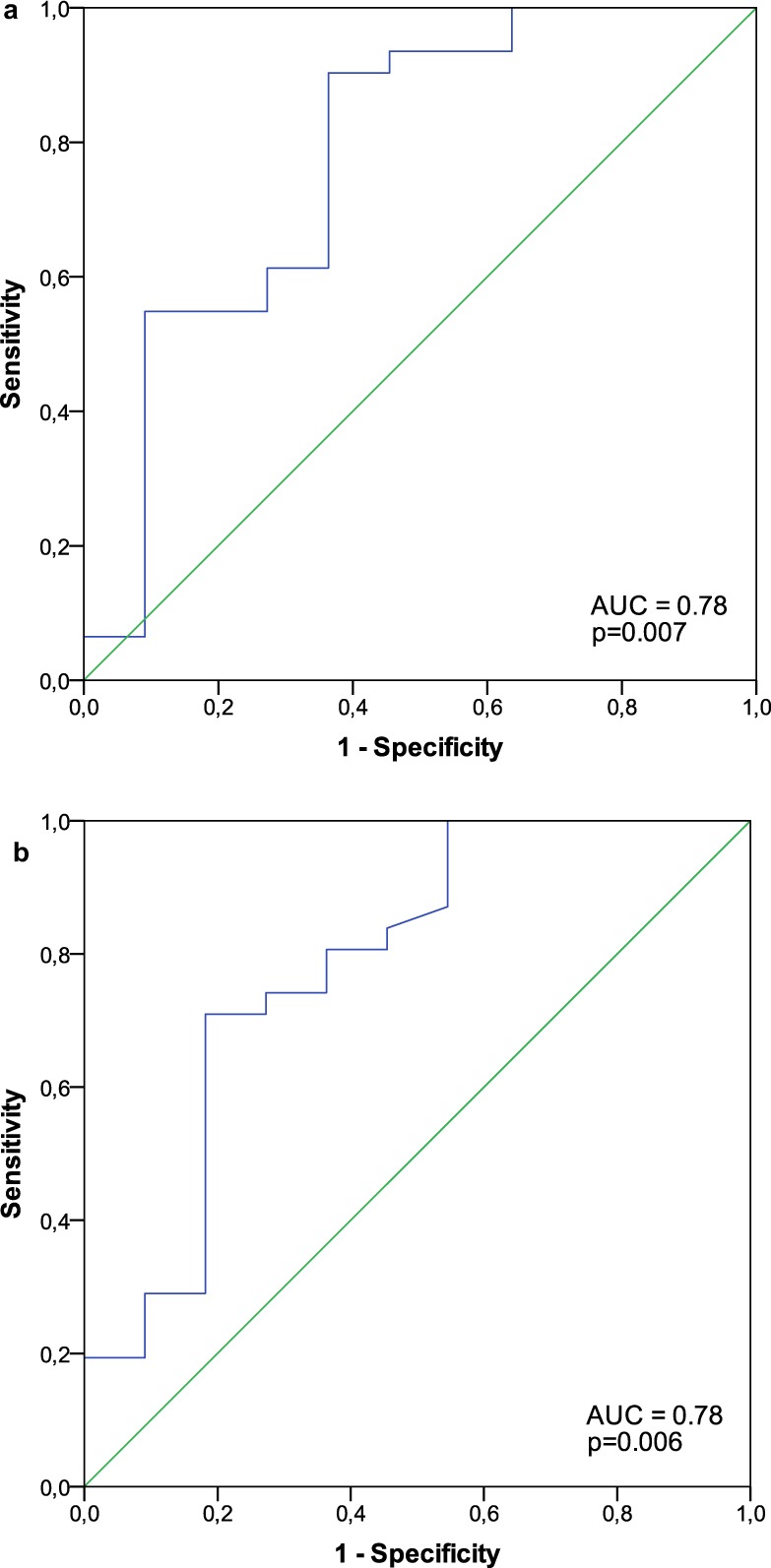
Receiver-operating characteristic (ROC) curve for the analysis of the predictive value of (**a**) functional capillary density: area under the curve (AUC) = 0.78 ± 0.09 (SE), CI 95%: 0.6–0.96, p = 0.007 and (**b**) total perfused capillary density: AUC = 0.78 ± 0.09 (SE), CI 95%: 0.61–0.96, p = 0.006, (depicted as blue line, respectively) for bleeding events. SE, standard error.

Areas under the ROC curves (AUC) for the analysis of the predictive value of capillary density for bleeding events were 0.78 for functional (p = 0.007; SE: 0.09 (SE), CI 95%: 0.6–0.96) and total perfused capillary density (p = 0.006; SE: 0.09, CI 95%: 0.61–0.96), respectively (Fig. 2a,b). ROC curve analysis revealed thresholds of 163/mm^2^ (specificity: 0.636, sensitivity: 0.903) for functional capillary density and 274/mm^2^ (specificity: 0.818, sensitivity: 0.71) for total perfused capillary density as best predictors of bleeding risk in VAD patients. However, capillary rarefaction was not related to all-cause mortality in VAD patients after a median follow up time of 42 months. In detail, 10 patients died and their functional capillary density was 192/mm^2^ (147–265/mm^2^) vs. 199/mm^2^ (166–297/mm^2^), p = 0.695 and total perfused capillary density was 261/mm^2^ (208–438/mm^2^) vs. 305/mm^2^ (242–499/mm^2^), compared to survivors, p = 0.406.

In addition, we analyzed the possible association of ischemic events and microcirculation. During the one-year follow up, 8 VAD patients suffered ischemic events. In detail, we observed 3 cases of pump thrombosis, 3 strokes, 1 transitory ischemic attack and 1 deep vein thrombosis with pulmonary embolism. There was no association between cumulative ischemic events and microcirculation (data in the supplement, Table 2).

### Associations with VAD parameters

The VAD parameters pulsatility index, waviness, flow, speed and power were correlated with microvascular parameters. No associations were found in these analyses (data not shown).

No differences in microvascular parameters between centrifugal- and axial-flow VAD systems could be found.

### Comparison of microvascular parameters between men and women

We observed 10% and 27% lower functional and total capillary densities in men with VAD support when compared to women. However, this finding was not significant, possibly due to the small number of patients (Supplementary Table 3). In the CHF group and in healthy controls there was no difference in capillary density between men and women (data not shown).

## Discussion

Our study is the first to provide data on *in vivo* microcirculation in CHF patients with mechanical circulatory support. These patients had significantly lower functional capillary density as compared to patients with CHF and guideline directed medical treatment alone, and rarefied functional and total capillary density as compared to healthy controls.

Despite ameliorated cardiac output by the mechanical circulatory support^1^, the initially expected improvement of functional microcirculatory capacity was not observed. Our present results show that VAD therapy might even have a further impact on perfused capillary density. This may also be due to initial impairment by heart failure – as earlier shown by our study group^15^, which cannot be restored by VAD- treatment. In addition, VAD patients had a pump flow of 5.2 L/min corresponding to almost normalized levels of cardiac output. Furthermore, VAD patients were characterized by comparable serum NT-proBNP levels (p = 0.053) and significantly lower creatinine levels. Both, NT-proBNP and creatinine levels, are currently regarded as the best markers of heart failure severity^21,22^.

It is known that chronic heart failure patients have rarefied myocardial capillary density, which is suggested to contribute to decreased coronary flow reserve^19^.

Skeletal muscle capillary rarefaction was initially observed in histopathological studies of patients with CHF through an invasive approach, sampling skeletal muscle biopsies^23–25^. The current study characterizes perfused capillary density by a non-invasive *in vivo* technique for the first time in patients with VAD treatment.

Microcirculatory alterations are suggested to be associated with shear-force induced AVWS evoking (gastrointestinal) bleeding events in VAD- treated patients: In 2000, Koscielny *et al*. described the occurrence of capillary torquation, dilatation and microscopic bleedings in patients with AVWS^11^. Recently, Geisen *et al*. investigated a large VAD patient cohort (n = 198), in which all patients developed AVWS^4^. Bleeding was shown to be the most adverse event occurring in almost 30% of the patients after VAD implantation^26–28^. Along with recurrent heart failure, gastrointestinal bleedings are the most common cause for post-operative re-admissions^28^. Gastrointestinal bleeding was initially observed as part of the Heyde syndrome in 1958 (aortic stenosis and gastrointestinal bleeding from angiodysplasia) and is today known to be evoked by the unfolding and loss of high molecular weight multimers of the vWF in AVWS^29^.

In our study, loss of functional and perfused total capillaries was associated with transfusion-requiring or life-threatening bleeding events within 1 year. This supports our hypothesis of systemic microcirculatory alterations and also provides new insight in the underlying pathophysiological processes, namely the rarefaction of perfused capillaries. However, the latter was not associated with overall death during long-term follow up.

The elevated functional/total perfused capillary ratio in VAD and CHF patients with standard medical treatment might reflect the body’s partial compensation corresponding to previous observations of perfused capillary recruitment in persons with an elevated cardiovascular risk profile^30^.

A limitation of our study is that residual confounding due to differences in demographic characteristics might be possible. In addition, we investigated a heterogeneous population of CHF patients and did not assess AVWS in our patient population. This was an explorative study, and though multiplicity adjustments were performed for group comparisons, statistics were not corrected for the multiple variables that were tested. Further, we did not routinely obtain echocardiographic measurements. Indeed, echocardiography is invaluable in the perioperative management of VAD implantation and further monitoring of right ventricular size and function as well as tricuspid or aortic valve regurgitation, VAD malfunction (mostly due to thrombus formation), hypovolemia, cardiac tamponade or pulmonary embolism^31^.

However, the left ventricular (LV) ejection fraction cannot be validly obtained during LVAD function due to non-physiological LV unloading^31^. However, we determined NT-proBNP levels, which are established prognostic markers^21^, and were comparable in the CHF groups.

The study was of observational character with a hypothesis generating aspect, therefore measurements were performed only after VAD implantation. The absence of measurements before VAD implantation is the most important limitation of our study. Therefore, it cannot be clearly differentiated between VAD induced capillary rarefaction or impairment induced by CHF itself. Significant differences in functional capillary density and perfused total capillaries between CHF patients and healthy volunteers point towards an effect of CHF on microvascular perfusion regardless of VAD therapy. [3] Patients with VAD might have been in a worse stage than the control group prior to implantation. However, at index time VAD patients had similar NT-proBNP to and lower creatinine levels than CHF patients without VAD support. Therefore, one may speculate that improvement of cardiac function by VAD does not reverse microcirculatory alterations. The mechanisms of altered microcirculation in CHF and its potential pharmacological modulation warrant further investigation and sequential measurements.

## Conclusion

Patients with end-stage heart failure on VAD therapy had substantially lower *in vivo* functional capillary densities as measured by videomicroscopy than healthy subjects and CHF patients without mechanical circulatory support, respectively. This was associated with bleeding events during one- year follow up.

## Supplementary information

Functional capillary impairment in patients with ventricular assist devices
