# Supplementary material for: Functional capillary impairment in patients with ventricular assist devices
Source: Sci Rep. 2019 Apr 11;9:5909. doi: 10.1038/s41598-019-42334-3 (PMC6459831; doi:10.1038/s41598-019-42334-3)
Supplement: Supplementary file 1 — Functional capillary impairment in patients with ventricular assist devices [file 41598_2019_42334_MOESM1_ESM.docx]

**Supplementary Information:**

# Functional capillary impairment in patients with ventricular assist devices

## Authors:

Patricia P. Wadowski, Barbara Steinlechner, Daniel Zimpfer, Thomas Schlöglhofer, Heinrich Schima, Martin Hülsmann, Irene M. Lang, Thomas Gremmel, Renate Koppensteiner, Sonja Zehetmayer, Constantin Weikert, Joseph Pultar, Bernd Jilma

**Supplementary Table 1: Medication in VAD and CHF patients**

| **Medication in** | **VAD patients** | **CHF patients** |
| --- | --- | --- |
|  | **Ordered by frequency n (%)** | |
| **Oral anticoagulation** | 42 (100) | 20 (49) |
| **Beta-Blockers** | 39 (93) | 41 (100) |
| **Acetylsalicylic acid** | 36 (86) | 16 (39) |
| **Furosemide** | 30 (71) | 28 (68) |
| **Angiotensin converting enzyme-inhibitors** | 27 (64) | 33 (81) |
| **Aldosterone receptor antagonists** | 22 (52) | 29 (71) |
| **Statins** | 22 (52) | 12 (29) |
| **Amiodarone** | 11 (26) | 5 (12) |
| **Angiotensin receptor blockers** | 7 (17) | 9 (22) |
| **Alpha-Blockers** | 3 (7) | 7 (17) |
| **Xipamide** | 2 (5) | 4 (10) |
| **Ivabradin** | 1 (2) | 7 (17) |
| **Thiazide diuretics** | 1 (2) | 3 (7) |
| **Cardiac glycosides** | 0 | 3 (7) |
| **Calcium-Antagonists** | 0 | 4 (10) |

**Supplementary Table 2: Ischemic events in VAD patients**

|  | **Ischemic events (n=8)** | **No ischemic events**  **(n=34)** | **p-value** |
| --- | --- | --- | --- |
| **PBR (µm)** | 1.88 (1.72 -2.09) | 1.93 (1.77 -2.06) | p=0.671 |
| **RBC Filling (%)** | 77 (73 -79) | 72 (70 - 77) | p=0.167 |
| **Perfused capillary density (n/mm^2^)** | 185 (142- 245) | 196 (166 - 300) | p=0.352 |
| **Total capillary density (n/mm^2^)** | 300 (221 -467) | 284 (238 - 473) | p=0.814 |
| **Ratio (%)** | 68 (43-77) | 67 (61 - 74) | p=0.888 |

Supplementary Table 2: Data are presented as median and IQR.

**Supplementary Table 3: Microcirculatory parameters in men and women with VAD support**

|  | **Males (n=33)** | **Females (n=9)** | **p-value** |
| --- | --- | --- | --- |
| **PBR (µm)** | 1.9 (1.77 - 2.03) | 2.04 (1.57 – 2.14) | p=0.546 |
| **RBC Filling (%)** | 73 (70 - 77) | 77 (70 - 79) | p=0.414 |
| **Perfused capillary density (n/mm^2^)** | 188 (154 -263) | 208 (177 - 311) | p=0.289 |
| **Total capillary density (n/mm^2^)** | 275 (228 - 464) | 376 (255 - 499) | p=0.249 |
| **Ratio (%)** | 69 (59 - 74) | 65 (61 - 82) | p=0.928 |

Supplementary Table 3: Data are presented as median and IQR.

**Quartiles:**

Functional capillary density:

1^st^ quartile: ≤ 164.5 /mm^2^

2^nd^ quartile: > 164.5 /mm^2^ - ≤ 196 /mm^2^

3^rd^ quartile: > 196/mm^2^ - ≤ 286/mm^2^

4^th^ quartile: > 286/mm^2^

Total perfused capillary density:

1^st^ quartile: ≤ 228/mm^2^

2^nd^ quartile: > 228/mm^2^ - ≤ 284/mm^2^

3^rd^ quartile: > 284/mm^2^ - ≤ 472.75/mm^2^

4^th^ quartile: >472.75/mm^2^
